# ﻿Resurrection of *Sorbustapashana* (Rosaceae) based on molecular and morphological evidence

**DOI:** 10.3897/phytokeys.247.132538

**Published:** 2024-10-03

**Authors:** Xin Chen, Wen-Xiang Hou, Jun-Ling Hu, Meng-Die Dong, Bao-Mei Tan

**Affiliations:** 1 Co-Innovation Center for Sustainable Forestry in Southern China, College of Life Sciences, Nanjing Forestry University, Nanjing, China Nanjing Forestry University Nanjing China

**Keywords:** Morphological data, plastome, *Sorbus*, synonym, taxonomy

## Abstract

*Sorbustapashana* (Rosaceae) from Taibai Shan, Qinling, China, has been treated as a synonym of *S.tianschanica*. Both species belong to a distinctive group characterized by white tomentose buds, relatively large flowers, and red fruits. However, these two species do not cluster together in the plastome-based phylogenetic analysis. Morphologically, *S.tapashana* differs from *S.tianschanica* by its persistent white tomentose on the peduncle, pedicels, rachis, both sides of the midrib on abaxial surface, its leaflets with 31–51 teeth on each side and much smaller corymbs and fruits. Therefore, *S.tapashana* is reinstated as a distinct species here.

## ﻿Introduction

*Sorbus* L. sensu stricto (Maleae, Rosaceae) comprises approximately 90 species of trees and shrubs (Phipps et al. 1990; McAllister 2005). The genus is confined to the Northern Hemisphere, with its distribution spanning Europe, Asia, and northern North America (McAllister 2005). Morphologically, *Sorbus* s.s. can be distinguished from other genera in Maleae by its imparipinnate leaves and relatively small fruits with persistent sepals and styles. This genus exhibits high diversity in China, particularly in the southwestern mountainous regions (Lu and Spongberg 2003).

The monophyly of *Sorbus* s.s. has been confirmed by recent phylogenetic studies (Campbell et al. 2007; Liu et al. 2019, 2020, 2022, 2023a, 2023b; Jin et al. 2023, 2024). However, the circumscription of species within this genus remains unclear, particularly for those native to China (Lu and Spongberg 2003; McAllister 2005). For instance, there is debate regarding the taxonomic status of *Sorbustapashana* C.K.Schneid. (Schneider 1906). This species has been recognized in numerous floristic and taxonomic works (Yü and Lu 1974; Gabrielian 1978; Phipps et al. 1990; Lu and Spongberg 2003) before being treated as a synonym of *S.tianschanica* Rupr. (Ruprecht 1869) by McAllister (2005). McAllister proposed that *S.tapashana* and *S.tianschanica* were conspecific, despite differences such as the denser white hairs on buds and leaflets of *S.tapashana*. However, based on our examinations of the protologues (Ruprecht 1869; Schneider 1906), type specimens, and specimens collected from the type localities, we hypothesize that *S.tapashana* may be a distinct species.

In this study, we integrate phylogenetic inference with extensive morphological data to reevaluate the relationship between *Sorbustapashana* and *S.tianschanica*. Our aims are to: (1) determine whether *S.tapashana* and *S.tianschanica* represent two distinct species, and (2) assess the phylogenetic relationships among *S.tapashana*, *S.tianschanica*, and other species within the genus *Sorbus* s.s.

## ﻿Materials and methods

### ﻿Taxon sampling

Leaf samples of *Sorbustapashana* (China, Shaanxi, Taibai Shan, September 7, 2023, *Xin Chen 2255*, *2257*) and *S.tianschanica* (Xinjiang, July 10, 2020, *Wenhao Fan 1761*) were collected from their type localities, Taibai Shan and Tianshan, respectively. Voucher specimens are deposited in the Herbarium of Nanjing Forestry University (**NF**).

### ﻿DNA extraction, sequencing, and genome assembly

Whole genomic DNA was isolated from silica-gel dried leaves using a modified CTAB method (Doyle and Doyle 1987). Short-insert (150 bp) paired-end libraries were prepared for genome skimming using the Illumina HiSeq 4000 sequencing platform at Beijing Genomics Institute (BGI, Shenzhen, China). De novo assembly was performed using GetOrganelle v.1.7.5.3 (Jin et al. 2020) with *Torminalisglaberrima* (NC033975) as a reference. Genomes were annotated using the PGA program (Qu et al. 2019) with *S.tianschanica* (ON049666) as a reference. Annotation errors were manually verified and corrected using Geneious v.9.0.2 software (Kearse et al. 2012).

### ﻿Phylogenetic analysis

The plastome dataset alignment for this study includes 41 individuals representing 37 *Sorbus* s.s. taxa. This dataset comprises three newly sequenced samples: two from *S.tapashana* and one from *S.tianschanica*, alongside 38 accessions sourced from GenBank (www.ncbi.nlm.nih.gov/genbank). *Photiniaprionophylla* (Franch.) C.K.Schneid. was used as the outgroup for phylogenetic analyses. GenBank accession numbers utilized in this study are listed in Table 1.

**Table 1.** Taxon name and GenBank accession numbers for all individuals included in this study.

Taxon	Genbank accession number	Taxon	Genbank accession number
*Photiniaprionophylla* (Franch.) C.K.Schneid.	NC045355.1	*Sorbuspohuashanensis* (Hance) Hedl.	OP613257.1
*Sorbusaestivalis* Koehne	NC068530.1	*Sorbuspoteriifolia* Hand.-Mazz.	OR915972.1
*Sorbusalbopilosa* T.T.Yu & L.T.Lu	OR915913.1	*Sorbusprattii* Koehne	NC085635.1
*Sorbusamabilis* Cheng ex T.T.Yu & K.C.Kuan	MT357029.1	*Sorbusrandaiensis* (Hayata) Koidz.	NC085665.1
*Sorbusaucuparia* L.	OR915953.1	*Sorbusrehderiana* Koehne	OR915914.1
*Sorbuscalifornica* Greene	NC085651.1	*Sorbusrufopilosa* C.K.Schneid.	NC085638.1
*Sorbuscommixta* Hedl.	MK920288.1	*Sorbussambucifolia* (Cham. & Schltdl.) M.Roem.	NC085654.1
*Sorbusdecora* (Sarg.) C.K.Schneid.	NC085652.1	*Sorbussargentiana* Koehne	OR915977.1
*Sorbusdiscolor* (Maxim.) Maxim.	OR915986.1	*Sorbusscalaris* Koehne	NC085637.1
*Sorbusdumosa* House	NC085653.1	*Sorbusscopulina* Hough	NC085658.1
*Sorbushelenae* Koehne	NC068536.1	*Sorbussetschwanensis* (C.K.Schneid.) Koehne	NC046777.1
*Sorbushimalaica* Gabrieljan	NC085572.1	*Sorbussibirica* (Hedl.) Prain	NC085576.1
Sorbushupehensisvar.hupehensis C.K.Schneid.	NC068721.1	Sorbussitchensisvar.grayi (Wenz.) C.L.Hitchc.	OR897861.1
Sorbushupehensisvar.paucijuga (D.K.Zang & P.C.Huang) L.T.Lu	MT916771.1	*Sorbustapashana* C. K.Schneid.	PQ031218; PQ031219
*Sorbusinsignis* (Hook.f.) Hedl.	NC051947.1	*Sorbustianschanica* Rupr.	PQ031217
*Sorbuskiukiangensis* T.T.Yu	NC085636.1	*Sorbustianschanica* Rupr.	MK920289.1
*Sorbuskiukiangensis* T.T.Yu	OR915919.1	*Sorbustianschanica* Rupr.	ON049666.1
*Sorbusmacrantha* Merr.	NC085631.1	*Sorbustianschanica* Rupr.	OK375442.1
*Sorbusmicrophylla* (Wall. ex Hook. f.) Wenz.	NC085633.1	*Sorbusulleungensis* Chin S.Chang	MG011706.1
*Sorbusmunda* Koehne	NC062714.1	*Sorbusunguiculata* Koehne	MK814479.1
*Sorbusoligodonta* (Cardot) Hand.-Mazz.	NC085634.1	*Sorbuswilsoniana* C.K.Schneid.	OR915983.1

The plastid genome dataset was aligned using MAFFT v.7.388 (Katoh and Standley 2013) within Geneious v.9.0.2, followed by manual adjustments. Phylogenetic relationships were inferred using both maximum likelihood (**ML**) and Bayesian inference (BI). ML analyses, employing the GTR+G nucleotide substitution model, were estimated with RAxML v.8.2.10, with 100 runs and 1,000 bootstrap (**BS**) Ronquist and Huelsenbeck 2003replicates (Stamatakis 2014). BI analyses were performed using MrBayes v.3.2.7 (Ronquist and Huelsenbeck 2003), running the Markov chain Monte Carlo (MCMC) for 2,000,000 generations with trees sampled every 1000 generations. The resulting trees from ML and BI analyses were visualized using FigTree v.1.4.3 (http://tree.bio.ed.ac.uk/software/figtree).

### ﻿Morphological analysis

Morphological characters were examined using our specimens and online images from various sources including herbaria A, HNWP, IBK, IBSC, IFP, KUN, LBG, NAS, NWTC, PE, and XBGH. These images were accessed through the Chinese Virtual Herbarium (http://www.cvh.ac.cn/), JSTOR Global Plants (https://plants.jstor.org/), the Global Biodiversity Information Facility (GBIF; https://www.gbif.org/), and the Plant Photo Bank of China (PPBC; http://ppbc.iplant.cn/). Measurements were taken from both actual specimens and those with scale bars in the images.

## ﻿Results and discussion

### ﻿Phylogenetic analyses

**Figure 1.**
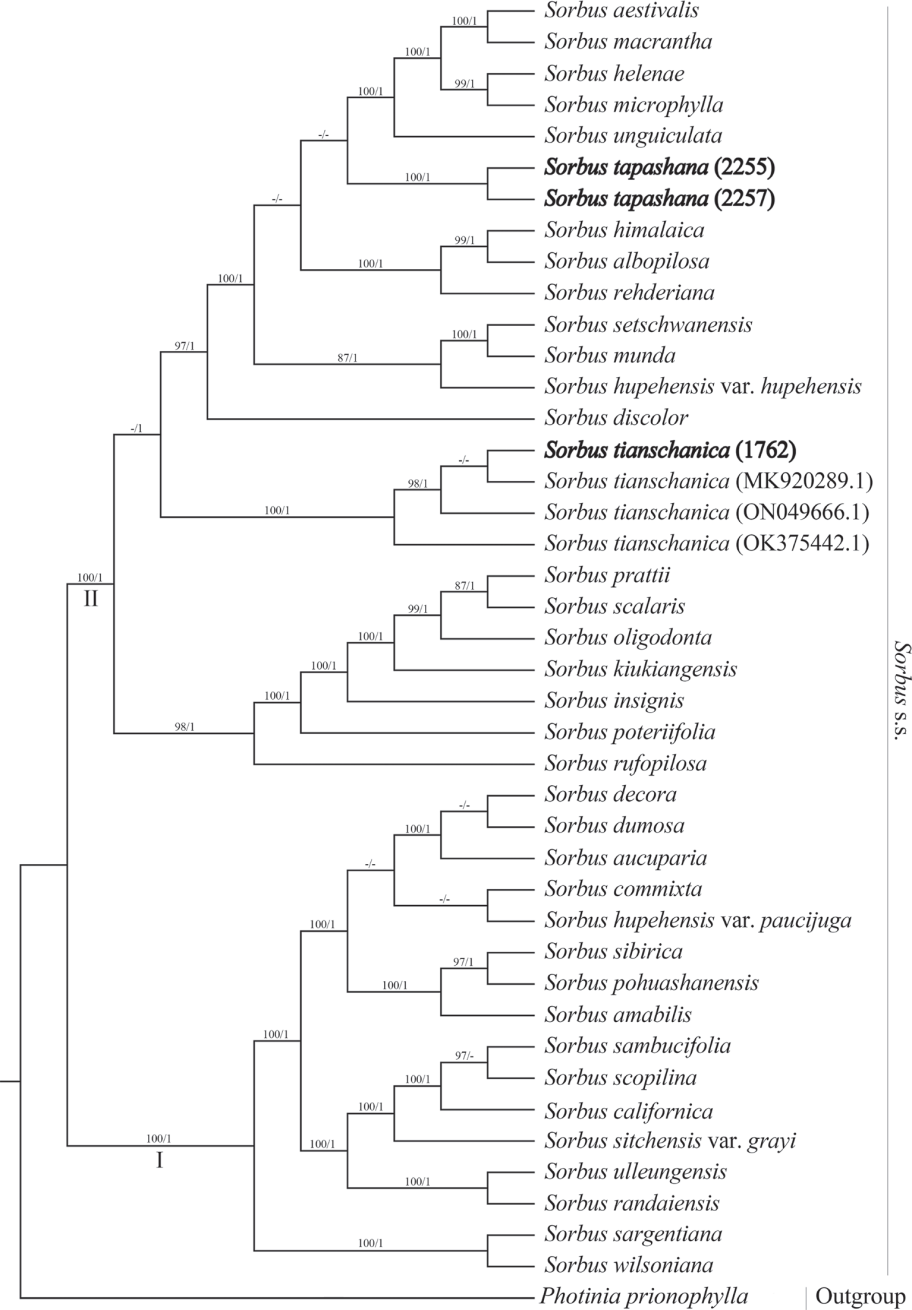
Phylogenetic tree of *Sorbus* s.s. resulting from the maximum likelihood analysis and Bayesian inference of the plastome data set. Numbers below the branches indicate bootstrap values (≥70%) of the ML analyses and the posterior probability (≥0.95) of Bayesian analyses.

The phylogenetic trees inferred from ML and BI methods were topologically congruent, with only minor differences in support values. Consequently, only the ML tree is presented here, with support values from both ML and BI analyses indicated at each node (Fig. 1).

Our results strongly support the monophyly of *Sorbus* s.s., which is divided into two major clades (Fig. 1: I and II). Clade I includes 14 species and two varieties within S.subg.Sorbus, excluding S.hupehensisvar.paucijuga (D.K.Zang & P.C.Huang) L.T.Lu. Clade II contains *S.tianschanica* and species from S.subg.Albocarmesinae McAll., with *S.tapashana* deeply nested within it. Notably, *S.tapashana* is distantly related to *S.tianschanica*.

Our findings unequivocally demonstrate the existence of two distinct, well-resolved clades within the monophyletic *Sorbus* s.s., which broadly align with the two subgenera proposed by McAllister (2005). However, contrary to previous classifications that placed *S.tianschanica* within subg. Sorbus due to its uniformly red fruits, our data show that it is embedded in clade II along with species from subg. Albocarmesinae, consistent with other recent molecular studies (Li et al. 2017; Tang et al. 2022; Wang et al. 2024). While the plastome dataset analyses show that *S.tapashana* and *S.tianschanica* are located in the same clade, they are isolated in different groups. *Sorbustapashana* is more closely related to *S.aestivalis* Koehne, *S.macrantha* Merr., *S.helenae* Koehne, *S.microphylla* (Wall. ex Hook.f.) Wenz., and *S.prattii* Koehne than to *S.tianschanica*. Given that monophyly is a widely accepted as criterion for taxonomic classification (Chiarini et al. 2022; Böhnert et al. 2023), *S.tapashana* should be recognized as a distinct species.

### ﻿Morphological analyses

**Figure 2.**
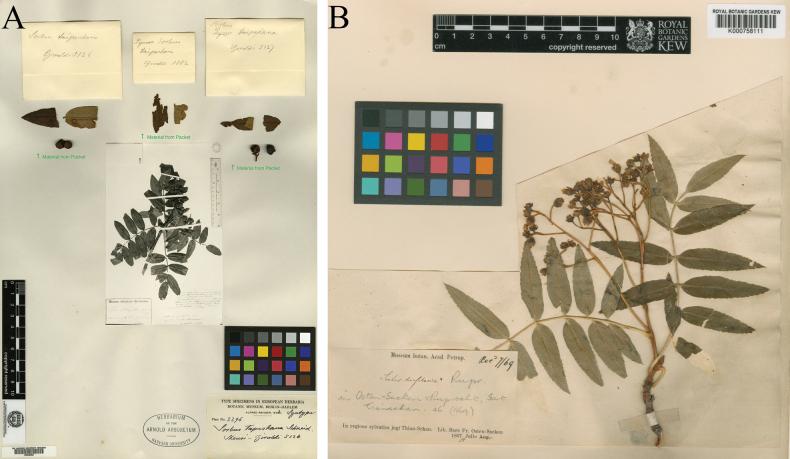
Lectotype of *Sorbustapashana* C.K.Schneid. (the upper left part of A00046062) and isotype of *S.tianschanica* Rupr. (K000758111).

**Figure 3.**
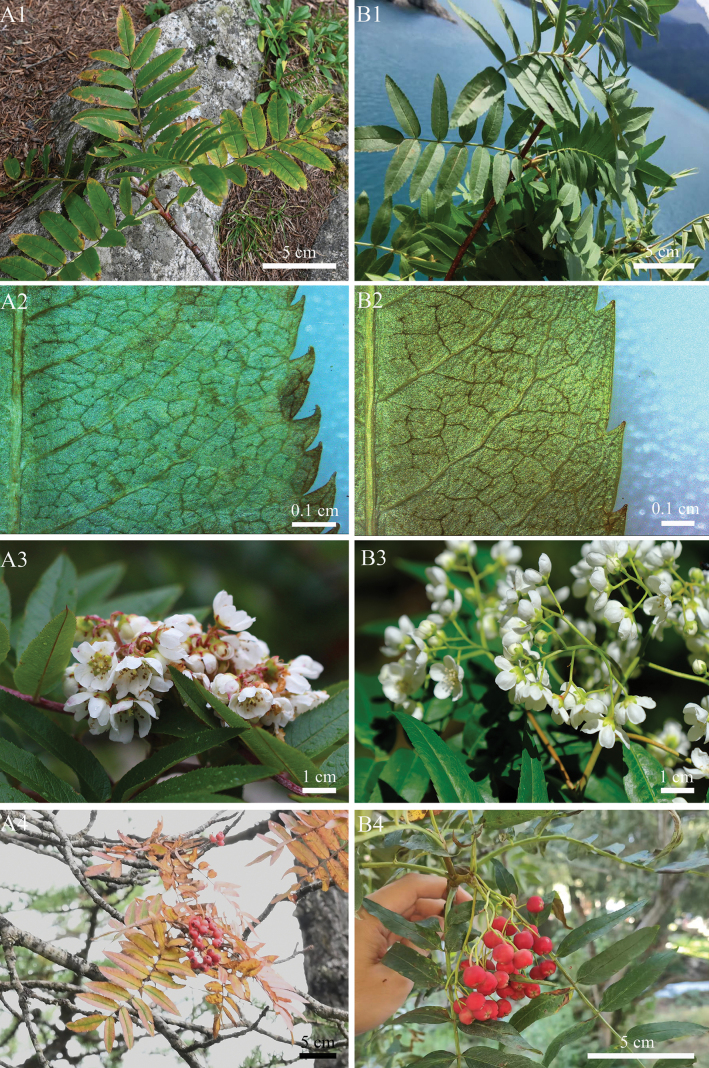
Comparison of morphological characters *Sorbustapashana* C.K.Schneid. (**A1–A4**) and *S.tianschanica* Rupr. (**B1–B4**) **1** sterile branch showing leaves and buds (**B2** were taken by Wenhao Fan) **2** leaflet margins **3** corymbs (**A3** and **B3** were taken by Renbin Zhu and Yongfu Xu, respectively) **4** fertile branch showing fruits (**B4** were taken by Wenhao Fan).

*Sorbustapashana* and *S.tianschanica* share several characters, including white tomentose winter buds, 5–7 pairs of leaflets, large flowers (1.5–2 cm in diameter), usually five and densely white tomentose styles, and red fruits (Table 2, Figs 2, 3). Consequently, both species were previously placed under ser. Tianschanicae Kom. ex T.T.Yü by Yü and Kuan (1963), Gabrielian (1978), and Phipps et al. (1990). Later, McAllister (2005) merged them as a single species. However, *S.tapashana* can be distinguished from *S.tianschanica* by several morphological characters (Table 2), e.g., more densely serrate leaflets (31–51 teeth per side compared to 12–24 teeth in *S.tianschanica*; Fig. 3: A2, B2), persistent white tomentose on the peduncle and pedicels (vs. sub-glabrous in *S.tianschanica*; Fig. 3: A3, B3), smaller inflorescences (5–9 × 5–8 cm compared to 6–10 × 7–12(–15) cm), and smaller fruits (8–10 mm vs. 10–12 mm in diameter; Fig. 3: A4, B4). Additionally, *S.tapashana* is restricted to Gansu and Shaanxi, China, whereas *S.tianschanica* has a much broader distribution area (Table 2).

**Table 2.** Comparison of morphological characters, phenologies, and distributions of *Sorbustapashana* and *S.tianschanica*.

	*Sorbustapashana*	*Sorbustianschanica*
Branchlets	brownish or grayish brown, terete, lenticellate	
Buds	white pubescent	
Leaves	9–18 cm in length	14–17 cm in length
Petiole	1.5–4.1 cm long	1.5–4.3 cm long
leaflets	(4–)5–7 pairs	(4–)6–7 pairs
The number of teeth on each side of the leaflet	31–51	12–24
Stipules	linear-lanceolate, 5–7 mm, caducous	linear-lanceolate, 7–11 mm, caducous
Inflorescences	5–9 × 5–8 cm, with persistent white pubescent	6–10 × 7–12(–15) cm, sub-glabrous
Flowers	1.5–2 cm in diameter	1.5–1.8(–2.0) cm in diameter
Styles	usually 5, densely white tomentose basally	(3–)5, densely white tomentose basally
Fruit	red, globose, 8–10 mm in diameter	scarlet, globose, 1–1.2 cm in diameter
Phenology	flowering in June; fruiting in September	flowering in May and June, fruiting in September and October
Distribution	Gansu, Shaanxi	Afghanistan, China (Gansu, Qinghai, and Xinjiang), Kazakhstan, Kirgizstan, Pakistan, Tadzhikistan, and Uzbekistan.

### ﻿Taxonomic treatment

#### ﻿ Sorbus tapashana

Taxon classificationPlantaeRosalesRosaceae

C.K.Schneid. in Bull. Herb. Boissier, sér. 2, 6: 313 (1906)

B9232BAB-B0D6-53C6-B083-98190D4D1C8B

≡ Pyrustapashana (C.K.Schneid.) M.F.Fay & Christenh. in Global Fl. 4: 123 (2018).

##### Type.

China • Shaanxi: the summit of Taibai Shan, 10–20 September 1897, *Giraldi 5126* (lectotype, designated here: A[A00046062 (the upper left part)] image!).

##### Note.

*Sorbustapashana* was first described by Schneider (1906). It was later transferred to the genus *Pyrus* L. s.l. along with other species from *Sorbus* sensu lato by Christenhusz et al. (2018). However, *Pyrus* s.l. has proven to be overly inclusive and polyphyletic (Liu et al. 2019, 2022; Tang et al. 2022; Jin et al. 2024; Wang et al. 2024).

When *Sorbustapashana* was described, Schneider (1906) cited three syntypes, *Giraldi 5126*, *5127*, and *1082*, deposited in the Berlin Herbarium (B). However, none of them could be located at B. In the herbarium of the Arnold Arboretum, Harvard University (A), duplicates of these gatherings are present. At A, we found the original materials of these gatherings on Plate no. 2296. The sheet, with barcode A00046062 (image available at: HUH - Databases - Specimen Search (harvard.edu)), bears a photo of specimen “Giraldi n. 5126” at B, and three fragments belonging to *Giraldi 5126*, *5127* and *1082* respectively (Fig. 1A). The photo of *Giraldi 5126* is a fruit specimen, with scale bars and three labels on it. One of the three is the determination label of Schneider, with “*Sorbustapashana*, an var. *S.pohuashanensis*?” on it, the same as those that had been stated in the protologue. *Giraldi 5126* is a good candidate to serve as lectotype because: (1) there is a photo of complete specimen from herbarium B which bears Schneider’s annotation; (2) the fragmentary material of *Giraldi 5126* has a leaflet (clearly showing the white tomentose persistent along the mid-vein abaxially) and two fruits. Therefore, we designate *Giraldi 5126* (the upper left part of A00046062) as the lectotype for the name.

In the protologue, it is implied that *Sorbustapashana* may be a variety of *S.pohuashanensis* (Hance) Hedl. However, it is distinguished from the latter in having much larger flowers (1.5–2 cm in diameter), deciduous linear-lanceolate stipules (vs. relatively small flowers 5–8 mm in diameter, persistent broadly ovate or semi-orbicular stipules). Furthermore, *S.tapashana* is resolved in clade II while *S.pohuashanensis* in subclade I. Therefore, *S.tapashana* and *S.pohuashanensis* are separate species.

## Supplementary Material

XML Treatment for Sorbus tapashana

## ﻿Additional information

### Author ORCIDs

Xin Chen https://orcid.org/0000-0003-2315-083X

Wen-Xiang Hou https://orcid.org/0000-0001-6928-5321

Jun-Ling Hu https://orcid.org/0009-0007-4527-0767

Meng-Die Dong https://orcid.org/0009-0004-1932-4950

Bao-Mei Tan https://orcid.org/0009-0000-8582-2780

### Data availability

All of the data that support the findings of this study are available in the main text.
